# Perception of Facial Profile: How You Feel About Yourself

**DOI:** 10.5005/jp-journals-10005-1092

**Published:** 2010-04-15

**Authors:** Mridula Trehan, Zuber Ahamed Naqvi, Sunil Sharma

**Affiliations:** 1 Professor and Head, Department of Orthodontics and Dentofacial Orthopedics, Mahatma Gandhi Dental College and Hospital, Jaipur, Rajasthan, India; 2 Senior Lecturer, Department of Orthodontics and Dentofacial Orthopedics, Mahatma Gandhi Dental College and Hospital Jaipur, Rajasthan, India; 3 Principal, Mahatma Gandhi Dental College and Hospital, Jaipur, Rajasthan, India

**Keywords:** Facial profile, Perception, Esthetics.

## Abstract

**Objective:**

The aim of this study was to determine how aware the individuals were of their own profile and to compare the orthodontist’s perception of an attractive facial profile with those of laypeople, dental students and orthodontic patients.

**Materials and methods:**

The study comprised of a total of 200 subjects divided into four groups of 50 subjects each: Laypeople, first-year dental students, final-year dental students and orthodontic patients. Participants answered a questionnaire regarding facial profile and their expectations from orthodontic treatment. The facial profile photographs of participants were analyzed by two orthodontists separately who matched the individual to the depicted silhouettes. Agreement between participants and orthodontists was evaluated by using the statistic χ^2^ test.

**Results:**

Dental students and orthodontic patients were more aware of their facial profile as compared to the laypeople. The four groups were different in their abilities to recognize their own profiles. The difference in profile perception between orthodontists and subjects was statistically significant (p < 0.05).

**Conclusions:**

Class I profiles were perceived to be the most attractive by all the groups and profiles with a protrusive mandible were perceived to be the least attractive. Final-year dental students and orthodontic patients were more accurate in identifying their own profile.

## INTRODUCTION

A good face enhances the self confidence of a person. An unesthetic face can affect the person physically, mentally, psychosocially and emotionally causing a deep dent in his/ her self-confidence.

Profile plays an important role in the treatment plan as it shows the anteroposterior position of jaws, lip posture, lip prominence, vertical facial proportions and mandibular plane angle. Hence, the technique of facial profile analysis has sometimes been called the “poor man’s cephalometric analysis”.^1^

Attractive adults and children are judged more favorably and treated more positively than unattractive adults and children, even by those who know them.^2^ People with attractive faces are regarded socially as more competent, successful and likeable.^2-4^ It has been shown that facial and dental anomalies that are sufficient to affect a person’s appearances might put that person at a social disadvantage.^5-6^

Hence, nowadays people seek orthodontic treatment to achieve pleasing esthetic facial profiles but the patient’s perception of an attractive facial profile may differ from that of an orthodontist’s perception.

Hence, the study was undertaken to determine how aware the individuals are of their own profile and to compare the orthodontist’s perception of an attractive facial profile with those of laypeople, dental students and orthodontic patients.

## MATERIALS AND METHODS

The study comprised of a total of 200 subjects divided into four groups of 50 subjects each:

*Group I:* First-year dental students
*Group II:* Laypeople
*Group III:* Final-year dental students
*Group IV:* Orthodontic patients

Residents of Jaipur, ranging in ages from 14 to 22 years were included in this study. The selection of the subjects was based on absence of any apparent facial deformities, syndromes affecting facial morphology and change in facial morphology due to trauma or psychological problems. Orthodontic patients were selected from the Department of Orthodontics and Dentofacial Orthopedics, Mahatma Gandhi Dental College and Hospital, Jaipur. First- and final-year students were also selected from the same college.

**Fig. 1:**
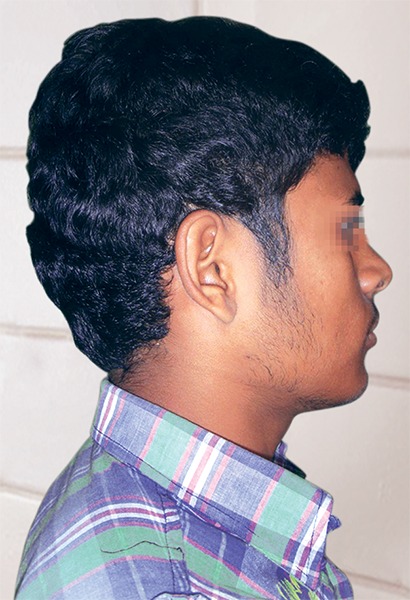
Profile photograph of subject

**Figs 2A to D:**
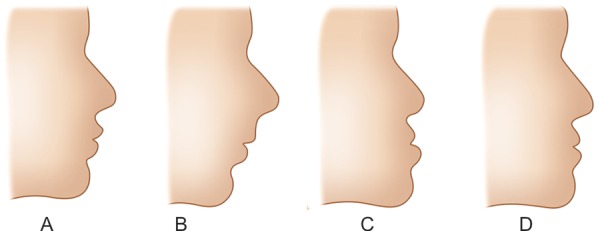
Silhouettes representing (A) class I (B) class II (C) class III (concave) and (D) straight profile

Individuals were given a questionnaire consisting of 10 questions regarding facial appearance. They were asked if they had ever noticed the profile (side view) of any person’s face and if yes, then which profile did they find the most attractive (Fig. 1), whether the profile contributes to facial beauty and which structures contribute the most to the profile. They were also asked to choose from various silhouettes (Figs 2A to D), the one they thought most resembled their own profile and if they were willing to undergo orthodontic treatment to change their profile. The facial profile of each participant was evaluated by two orthodontists separately.

## RESULTS

Differences among the groups and agreement for facial profile between participants and orthodontists were evaluated by Chi-square test (χ^2^). The statistical significance level was set at p < 0.05 for the statistical analysis.

**Graph 1:**
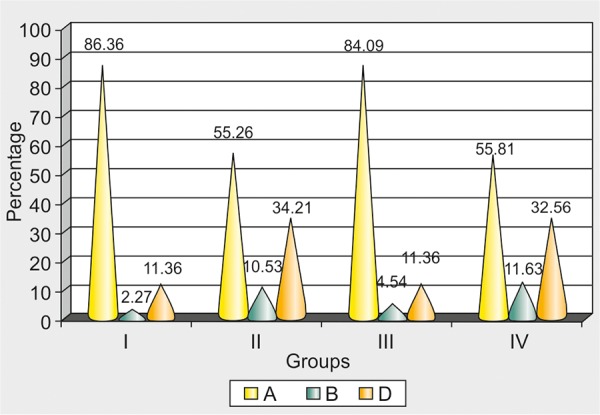
Distribution of most attractive profiles

The profile found to be the most attractive by the subjects was class I (71%) followed by straight profile (22%) and class III profiles were found to be the least attractive (0%) (Table 1 and Graph 1).

Almost all the subjects felt that profile contributes to facial beauty.

**Graph 2:**
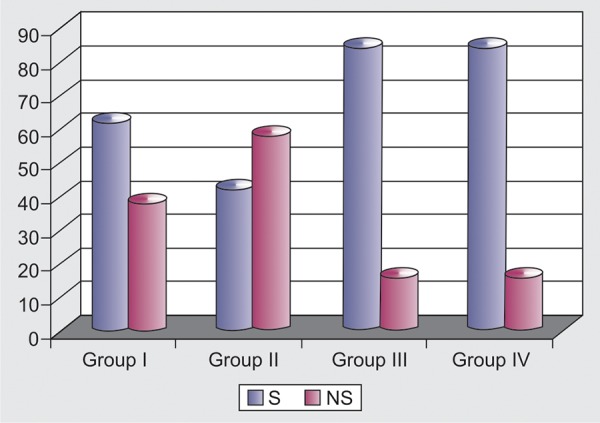
Agreement of profile judgement

The four groups were different in their ability to perceive their own profile. Final-year dental students and orthodontic patients were most accurate in identifying their own profiles (84%). This was followed by first-year dental students (62%) and laypeople (42%). There is statistically significant difference in the agreement between laypeople and first-year dental students perception of their own profiles and evaluation by orthodontists (p < 0.05) (Table 2 and Graph 2).

## DISCUSSION

Professional assessment of dental appearance is important, but patient’s opinions regarding dental appearance should also be respected and included in assessments for treatment planning.^7^ Hence, this study was conducted to find out the perception of profile by different population groups. A similar study was conducted on the Chinese population to assess the perception of Chinese facial profile esthetics.^8^ Another study was done for analysis of the soft tissue facial profile of Croatians using linear measurements.^9^

Previous studies have shown that concepts of esthetics are influenced by the level of dental or speciality training,^10,11^ and orthodontic patients might have become educated during the initial consultation and might have increased expectations because of the treatment that they were receiving.^11^ The same can be applied to final-year dental students as well.

**Table 1:** **Table 1:** Distribution of most attractive profile according to various group subjects

*Groups*		*Most attractive profile*						*Total*	
		*A*		*B*		*D*			
I		38 (86.36)		1 (2.27)		5 (11.36)		44 (100.00)	
II		21 (55.26)		4 (10.53)		13 (34.21)		38 (100.00)	
III		37 (84.09)		2 (4.54)		5 (11.36)		44 (100.00)	
IV		24 (55.81)		5 (11.63)		14 (32.56)		43 (100.00)	

**Table 2:** **Table 2:** Agreement on profile as judged by orthodontist and subjects

*Groups*		*Results*				*Total*	
		*Similar*		*Not Similar*			
I		31 (62.00)		19 (38.00)		50 (100.00)	
II		21 (42.00)		29 (58.00)		50 (100.00)	
III		42 (84.00)		2 (16.00)		50 (100.00)	
IV		42 (84.00)		3 (16.00)		50 (100.00)	

The study showed that 86.36% of first-year dental students and 84.09% of final-year dental students found class I profiles to be the most attractive. This is somewhat similar to the findings of Jen Soh et al^8^ who conducted a study on comparative assessment of perception of Chinese facial profile and reported normal profiles to be the most attractive. This finding can be of use in deciding the orthodontic treatment plan which results in a change in the facial profile as different populations like different types of facial profile.

It was found that subjects were inaccurate in their perceptions of their own profiles. The most accurate judgements of profile were made by final-year dental students (84%) and orthodontic patients (84%), followed by the first-year dental students (62%) whereas the laypeople were the least accurate (42%). Dental students become more aware of esthetics during their dental education.^10^ Therefore, it is not surprising that this group was significantly different from group I and group II in identifying their profiles correctly. This result is in agreement with those of Eser Tufekci et al^12^ who found that the third-year dental students were most accurate in identifying their own profiles (64%) and laypeople were the least accurate (43%). This is somewhat expected as laypeople usually do not view themselves from the profile and, unless it is pointed out to them by a clinician, they may not be aware of their own profiles.

Hence, it is important for clinicians to be aware of how patients perceive their own appearance because failure in communication may result in patient dissatisfaction despite well-intentioned treatment planning on the part of the clinician.

## CONCLUSION

The following conclusions were obtained:

1. The subjects of all the groups perceived class I profiles to be the most attractive followed by straight profiles
2. The profiles with protrusive mandibles were perceived to be the least attractive by all the four groups
3. Among the four groups, final-year students and orthodontic patients were able to identify their profiles almost as accurately as the orthodontists.
